# A combination therapy for *KRAS*-mutant lung cancer by targeting synthetic lethal partners of mutant *KRAS*

**DOI:** 10.1186/s40880-016-0154-7

**Published:** 2016-10-28

**Authors:** Xiufeng Pang, Mingyao Liu

**Affiliations:** 1 Shanghai Key Laboratory of Regulatory Biology, Institute of Biomedical Sciences and School of Life Sciences, East China Normal University, 500 Dongchuan Road, Shanghai, 200241 P. R. China; 2 Department of Molecular and Cellular Medicine, Institute of Biosciences and Technology, Texas A&M University Health Science Center, 2121W. Holcombe Blvd., Houston, TX 77030 USA

**Keywords:** Synthetic lethality, KRAS, Polo-like kinase 1, RhoA/Rho kinase, Combination therapy

## Abstract

The *KRAS* gene is frequently mutated in multiple cancer types, but it fell off the drug discovery radar for many years because of its inherent “undruggable” structure and undefined biological properties. As reported in the paper entitled “Suppression of KRas-mutant cancer through the combined inhibition of KRAS with PLK1 and ROCK” in *Nature Communications*, we performed a synthetic lethal screening with a combinatorial strategy on a panel of clinical drugs; we found that combined inhibition of polo-like kinase 1 and RhoA/Rho kinase markedly suppressed tumor growth in mice. An increase in the expression of the tumor suppressor P21^WAF1/CIP1^ contributed to the synergistic mechanism of the combination therapy. These findings open a novel avenue for the treatment of *KRAS*-mutant lung cancer.

## Main text

*RAS* genes (*KRAS*, *HRAS*, and *NRAS*) act as guanosine triphosphate (GTP)/guanosine diphosphate (GDP)-regulated binary on–off switches, regulating diverse normal cellular functions. Reportedly, nearly one-third of human cancers exhibit aberrantly activated *RAS* mutations [1]. As a principal isoform of three *RAS* genes, *KRAS* is the “black sheep” in this family, and cancers related to mutations in *KRAS* (*KRAS4A* and *KRAS4B*) alone account for approximately one million deaths per year [2]. Although intensive studies have been conducted on K-Ras protein structure, biochemistry, signaling, and biology, effective treatments for *KRAS*-mutant cancers have not yet been developed. Recent discovery of KRAS^G12C^-selective inhibitors has invigorated the Ras community [3, 4]; however, these compounds are currently used as chemical probes to delineate K-Ras signaling rather than as therapeutic agents that can be translated directly to the clinic.

As an indirect approach to target mutant *KRAS*, synthetic lethality studies have found a novel way to block mutant K-Ras signaling [5]. Several synthetic lethal RNA interference screening studies have characterized a list of synthetic lethal interactors of mutant *KRAS* [6–8]. Clinical inhibitors targeting some of these synthetic lethal partners, such as the anti-apoptotic protein B cell lymphoma-extra large (BCL-XL), cyclin-dependent kinase 4 (CDK4), and serine/threonine-protein kinase TBK1, have already been tested in clinical settings in a combinatorial strategy with a mitogen-activated protein kinase (MEK) inhibitor to treat *KRAS*-mutant cancers [5]. These important achievements based on synthetic lethality encouraged us to perform a chemical screening in the hope of developing efficacious clinical regimens to benefit patients with *KRAS*-driven cancers. As reported in the paper entitled “Suppression of KRas-mutant cancer through the combined inhibition of KRAS with PLK1 and ROCK” [9] in a recent issue of *Nature Communications*, we developed a synthetic lethal drug screening with a combinatorial strategy using a panel of clinical agents, and we identified several unique genotype-selective synergistic drug pairs that selectively killed *KRAS*-mutant cells but spared untransformed ones. In addition, we systematically investigated the selective anti-RAS efficacy of the best combinatorial hit that inhibited both polo-like kinase 1 (PLK1) and RhoA/Rho kinase (ROCK).

In this study, we employed the primary screening in paired isogenic cell lines T29 and T29Kt1. Mutationally activated KRAS^G12V^ was constitutively expressed in T29Kt1 cells, which could form subcutaneous tumors after being injected into immunocompromised mice [10]. According to the assumption that tumor initiation, progression, and heterogeneity are primarily driven by multiple genetic disorders rather than by a single defect, a “cocktail” of drugs is often given to achieve more potent efficacy [11]. Therefore, we employed a combinatorial strategy on a panel of clinical agents targeting K-Ras signaling directly or indirectly, including K-Ras upstream pathways, K-Ras downstream pathways, or mutant K-Ras-dependent pathways. By analyzing the combinatorial efficacy of over 300 pairs of different drug combinations with a Fa-CI plot, we found that the unique drug pair of BI-2536 (a PLK1 inhibitor) and fasudil (a ROCK inhibitor) was among the most efficacious. Inhibition of *KRAS*-mutant tumors by using surrogate drugs already approved for clinical use results in the fastest translation to the clinic. In line with these findings, BI-2536 has been reported in phase II clinical trials to effectively inhibit different types of tumor [12], and fasudil has been approved in Japan and China for the treatment of cerebral vasospasm, stroke, and hypertension, indicating that the combination of these drugs has acceptable clinical toxicity and significant translational potential. Also, we used four untransformed cell lines and 28 cancer cell lines with different genotypes, including *KRAS* mutation, *NRAS* mutation, epidermal growth factor receptor (*EGFR*) mutation, and *KRAS* wild-type, to verify the synergistic effect of the synthetic lethal genes polo-like kinase 1 (*PLK1*) and RhoA/Rho kinase (*ROCK*). As expected, the selectivity and specificity of this combination toward *KRAS*-mutant cells were further revealed. Next, the Lox-Stop-Lox (LSL)-KRAS^G12D^ mouse model and a patient-derived tumor explant model of lung cancer carrying an activating *KRAS* ^G12D^ mutation were set up to evaluate the in vivo efficacy and toxicity of combining BI-2536 and fasudil. Mice were treated with clinically relevant doses of BI-2536 and fasudil by either intravenous injection or oral gavage, leading to a significant reduction of tumor growth and extended mouse survival. These results indicate that our synthetic lethal screening study, based on clinically available drugs and drug combinations, may be help to develop potential novel and efficacious treatments for recalcitrant *KRAS*-mutant lung cancer.

We used a microarray analysis and several cell function assays to define the potential targets of the combined inhibition of PLK1 and ROCK. The gene expression profiles in *KRAS*-mutant A549 cells treated with BI-2536 and fasudil showed that the p53 signaling pathway had the highest number of significantly up-regulated genes and was significantly involved in the sensitivity of this drug pair. To further examine the precise mechanism of action of this combination therapy, we detected the protein levels of P53 and its several downstream targets. This combination had little effect on P53 protein level; however, it acutely increased the level of the cyclin-dependent kinase (CDK) inhibitor P21^WAF1/CIP1^ in *KRAS*-mutant T29Kt1 cells but not in the isogenic wild-type T29 cells. Similar mechanism results were obtained in several *KRAS*-mutant lung cancer cell lines.

A large body of literature shows that the CDK inhibitor P21 functions as both a sensor and an effector of multiple anti-proliferative signals [13]. P21 is implicated in cell mitotic regulation owing to its ability to stabilize CDK-cyclin complexes; furthermore, it binds to and inhibits proliferative cell nuclear antigen through its carboxyl-terminal domain. In addition, we confirmed the underlying mechanism of the combined inhibition of PLK1 and ROCK using P21 total knockout cells generated by the CRISPR/CAS9 system. The results showed that total depletion of P21 led to a complete rescue of G_2_/M arrest mediated by the drug pair only in *KRAS*-mutant cancer cells, suggesting that P21 was unambiguously necessary for the combinatorial action. Because it has been suggested that mutations in *KRAS* contribute to chromosome instability and mitotic stress [14], we assumed that increased mitotic stress by activating P21^WAF1/CIP1^ would cause susceptibility to apoptosis in *KRAS*-mutant cells. Such speculation is consistent with the perception of the “non-oncogene addiction” property of cancer cells. Collectively, our mechanism study of the combined inhibition of PLK1 and ROCK revealed a new synthetic lethal interaction between *KRAS* and *CDKN1A* (encoding P21), as genetic or pharmacologic increase of P21^WAF1/CIP1^ level preferentially impairs the growth of *KRAS*-mutant cells.

## Conclusions

Our study demonstrated the clinical feasibility of exploring a synthetic lethal regimen of the combined inhibition of PLK1 and ROCK to defeat *KRAS*-mutant cancer through a mechanism that involves activation of tumor suppressor P21. *CDKN1A* is characterized as a new “synthetic lethal partner” of mutant *KRAS* by overloading cell mitosis stress, especially in *KRAS*-mutant cells. Finally, we showed that this combinatorial strategy, by targeting the synthetic lethal partners of the oncogene *KRAS*, is a potential novel and efficacious treatment of *KRAS*-mutant cancers. This combinatorial drug screening based on synthetic lethality may be similarly applied to other oncogenic drivers or tumor suppressors that have been generally thought to be “undruggable.”
